# Recombinant interleukin-21 plus sorafenib for metastatic renal cell carcinoma: a phase 1/2 study

**DOI:** 10.1186/2051-1426-2-2

**Published:** 2014-01-27

**Authors:** Shailender Bhatia, Brendan Curti, Marc S Ernstoff, Michael Gordon, Elisabeth I Heath, Wilson H Miller, Igor Puzanov, David I Quinn, Thomas W Flaig, Peter VanVeldhuizen, Kelly Byrnes-Blake, Jeremy A Freeman, Rachel Bittner, Naomi Hunder, Sonia Souza, John A Thompson

**Affiliations:** 1 University of Washington, Seattle, WA, USA; 2 Providence Portland Medical Center, Portland, OR, USA; 3 Norris Cotton Cancer Center, Dartmouth Hitchcock Medical Center, Lebanon, NH, USA; 4 Pinnacle Oncology Hematology, Scottsdale, AZ, USA; 5 Karmanos Cancer Institute, Wayne State University, Detroit, MI, USA; 6 Lady Davis Institute and Segal Cancer Centre, Jewish General Hospital, McGill University, Montreal, Quebec; 7 Vanderbilt-Ingram Cancer Center, Vanderbilt University, Nashville, TN, USA; 8 Norris Comprehensive Cancer Center, University of Southern California, Los Angeles, CA, USA; 9 University of Colorado Cancer Center, Aurora, CO, USA; 10 University of Kansas Medical Center, Westwood, KS, USA; 11 ZymoGenetics (Bristol-Myers Squibb), Seattle, WA, USA; 12 Formerly of ZymoGenetics (Bristol-Myers Squibb), Seattle, WA, USA; 13 Division of Medical Oncology, Department of Medicine, University of Washington, Seattle Cancer Care Alliance, 825 Eastlake Ave East, Mailstop G4-830, Seattle, WA 98109-1023, USA

**Keywords:** Interleukin-21, Sorafenib, Renal cell carcinoma (RCC), Immunotherapy, Cytokine, VEGF, Tyrosine kinase inhibitors (TKI), Durable response, Targeted therapy

## Abstract

**Background:**

Despite the positive impact of targeted therapies on metastatic renal cell carcinoma (mRCC), durable responses are infrequent and an unmet need exists for novel therapies with distinct mechanisms of action. We investigated the combination of recombinant Interleukin 21 (IL-21), a cytokine with unique immunostimulatory properties, plus sorafenib, a VEGFR tyrosine kinase inhibitor.

**Methods:**

In this phase 1/2 study, 52 mRCC patients received outpatient treatment with oral sorafenib 400 mg twice daily plus intravenous IL-21 (10–50 mcg/kg) on days 1–5 and 15–19 of each 7-week treatment course. The safety, antitumor activity, pharmacokinetic and pharmacodynamic effects of the combination were evaluated.

**Results:**

In phase 1 (n = 19), the maximum tolerated dose for IL-21 with the standard dose of sorafenib was determined to be 30 mcg/kg/day; grade 3 skin rash was the only dose-limiting toxicity. In phase 2, 33 previously-treated patients tolerated the combination therapy well with appropriate dose reductions; toxicities were mostly grade 1 or 2. The objective response rate was 21% and disease control rate was 82%. Two patients have durable responses that are ongoing, despite cessation of both IL-21 and sorafenib, at 41+ and 30+ months, respectively. The median progression-free survival in phase 2 was 5.6 months. The pharmacokinetic and pharmacodynamic properties of IL-21 appeared to be preserved in the presence of sorafenib.

**Conclusion:**

IL-21 plus sorafenib has antitumor activity and acceptable safety in previously treated mRCC patients. IL-21 may represent a suitable immunotherapy in further exploration of combination strategies in mRCC.

**Trial registration:**

ClinicalTrials.gov Identifier: NCT00389285

## Background

The advent of antiangiogenic therapies targeting the vascular endothelial growth factor (VEGF) pathway has changed the therapeutic landscape of mRCC [1-7]. However, the effectiveness of targeted agents appears to decrease beyond the first-line setting and complete remission (CR) remains rare. High-dose interleukin-2 (IL-2) has been associated with durable CR in a small subset of patients, but the therapeutic application of IL-2 is limited by treatment-associated toxicities and a lack of biomarkers predictive of responses to therapy [8]. Novel therapies with distinct mechanisms of action are needed to further advance patient-outcomes in mRCC.

Interleukin 21 (IL-21) is a class I cytokine produced by activated CD4+ T cells and natural killer T (NKT) cells [9]. IL-21 boosts antitumor immunity through modulation of adaptive as well as innate immune responses. Specifically, IL-21 stimulates expansion and cytotoxicity of CD8+ T cells [10], enhances T-cell dependent B-cell proliferation and antibody production [9], and facilitates differentiation and activation of NK cells [9,11]. Unlike interleukin-2 (IL-2), IL-21 renders CD4+ T cells resistant to regulatory T cell suppression and does not enhance proliferation of regulatory T cells [12,13]. IL-21 may also enhance antitumor memory T cell responses [14], and has been associated with angiostatic activity [15]. Antitumor effects of IL-21 have been observed in various murine cancer models and may be mediated by cellular and humoral immune responses [10,16-20].

Recombinant IL-21 therapy has been investigated in several human trials [21-23]. In a phase 1 trial [22], IL-21 monotherapy was administered daily in an outpatient setting to forty-three patients with melanoma (n = 24) or mRCC (n = 19) on days 1–5 (week 1) and 15–19 (week 3) of a 7-week treatment course. The maximum tolerated dose (MTD) of IL-21 monotherapy with this schedule was determined to be 30 mcg/kg. The most common adverse events included flu-like symptoms, pruritis and rash. Treatment was associated with dose-dependent increases in soluble CD25 (sCD25; IL-2R-α), which is cleaved from T and NK cells on activation [24]. The antitumor activity in 17 evaluable mRCC patients was promising with an objective response rate (ORR) of 21% (4/17), and a disease control rate (DCR) of 89%; the four patients with an objective response had either not received any prior systemic therapy (n = 2) or had been treated with cytokines (n = 2) [22].

The unique immunostimulatory properties, tolerability and antitumor activity of IL-21 in mRCC encouraged investigation of its use in combination with other emerging therapies for mRCC. At the time of conception of this trial, sunitinib and sorafenib, both VEGF-receptor tyrosine kinase inhibitors (VEGFR-TKI), had recently been approved by the United States (US) Food and Drug Administration (FDA) for treatment of mRCC. The distinct antitumor mechanisms of action of VEGFR-TKI and cytokines suggested potential increased efficacy with their use in combination compared to either agent alone [25]. Indeed, VEGFR-TKI’s have been associated with reversal of immune suppression in the tumor microenvironment through reduction of regulatory T-cells and myeloid-derived suppressor cells and this may enhance the efficacy of immunotherapeutic agents [26-28]. Similarly, immunomodulatory cytokines including IL-21 have been associated with antiangiogenic effects that may add to the efficacy of VEGFR-TKIs in mRCC [15]. Preclinical studies suggested that sorafenib, a VEGFR-TKI, does not inhibit the effects of IL-21 on CD4+ or CD8+ T cell proliferation, NK cell activation, or antibody-dependent cellular cytotoxicity; also, the IL-21 and sorafenib combination led to improved tumor shrinkage and survival time as compared to either therapy alone in the murine RenCa model [29].

This phase 1/2 clinical trial evaluated the safety, anti-tumor activity, pharmacokinetic and pharmacodynamic effects of the combination of IL-21 with sorafenib in patients with mRCC.

## Results

### Patients

Fifty-two mRCC patients were enrolled and treated in this study. The baseline characteristics of patients are shown in Table 1. Demographic characteristics of the study population were representative of RCC, with a median age >60 years and male preponderance [30]. The study patients were categorized as either low- or intermediate-risk by the Memorial Sloan Kettering Cancer Center (MSKCC) risk classification [31].

**Table 1:** Demographics and baseline characteristics (Phase 1 and 2)

**Parameter, n (%)**	**Category/statistic**		**Phase 1 (N = 19)**	**Phase 2 (30 mcg/kg; N = 33)**
Gender, n (%)	Male		15 (79)	26 (79)
	Female		4 (21)	7 (21)
Age in years, Median (range)			63 (48–77)	61 (46–75)
ECOG, n (%)	0		15 (79)	15 (45)
	1		4 (21)	18 (55)
Prognostic risk category*, n (%)	Low risk		12 (63)	17 (52)
	Intermediate risk		7 (37)	16 (48)
Prior systemic treatment regimens for mRCC**, n (%)	0		10 (53)	-
	1		8 (42)	25 (76)
	2		0 (0)	8 (24)
	3		1 (5)	
Agents used for prior systemic therapy**, n (%)	**VEGFR-TKIs**			
		Sunitinib	4 (21)	19 (58)
		Pazopanib		1 (3)
		Cediranib		1 (3)
	**mTOR Inhibitors**			
		Temsirolimus		5 (15)
		Everolimus		2 (6)
	**Bevacizumab**			3 (9)
	**Immunotherapy**			
		IL-2	4 (21)	11 (33)
		Interferon		1 (3)
		Vaccine		3 (9)
		TLR-9 agonist	1 (5)	
		IL-2 gene therapy	1 (5)	
	**Other**			
		Vinblastine		1 (3)
		ABT-510	1 (5)	

*Based on Memorial Sloan Kettering Cancer Center (MSKCC) risk categorization.

**Number of prior treatment regimens, could consist of a combination of agents.

Nineteen patients were treated in the phase 1 portion; approximately half of the patients had received prior systemic treatment. Thirty-three patients were enrolled in the phase 2 portion; all patients had received 1 or 2 prior systemic therapy regimens that included VEGF-receptor TKIs (VEGFR-TKIs), mammalian target of rapamycin (mTOR) inhibitors, bevacizumab and/or immunomodulatory therapies; each regimen could consist of a combination of multiple agents.

### Safety experience

#### Phase 1 (dose escalation)

Four dose levels of IL-21 were evaluated in combination with the standard dose of sorafenib: 10 mcg/kg (n = 8), 30 mcg/kg (n = 4), 50 mcg/kg (n = 4), and 40 mcg/kg (n = 3). Three patients who received, in violation of the protocol, either incorrect (n = 2) or insufficient (n = 1) dosing to allow adequate safety assessment at the planned doses were replaced by other evaluable patients. One patient in the 10 mcg/kg cohort experienced grade 3 skin rash; the cohort was expanded with no further DLTs. No DLT occurred in the 30 mcg/kg cohort. Two patients in the 50 mcg/kg cohort had grade 3 skin rashes as DLTs, and the cohort was closed. Although there were no protocol-defined DLTs at the 40 mcg/kg dose, all patients in this cohort required sorafenib dose reductions due to rash or hand-foot syndrome. Hence, 30 mcg/kg was determined to be the recommended Phase 2 dose of IL-21 in combination with sorafenib at the standard dose of 400 mg twice daily.

#### Phase 2

The common clinical and laboratory AEs observed in phase 2 patients treated with 30 mcg/kg IL-21 plus sorafenib (starting at 400 mg twice daily) are listed in Table 2. The majority of toxicities were grade 1 or 2. The most common clinical symptoms included fatigue, diarrhea, fever, chills, hand-foot syndrome (HFS), and skin rash. Many symptoms, including fever, chills, fatigue, nausea, and vomiting, were observed transiently during the weeks of IL-21 administration. The most common grade 3 or higher AEs were skin rash (29%), HFS (24%) and fatigue (9%). The skin rash was typically a generalized maculopapular erythematous eruption arising in the first two weeks of treatment and progressing rapidly. With prompt treatment-interruption, the rash typically resolved over a few days and most patients were able to resume and tolerate treatment at the reduced dose of sorafenib while maintaining the same dose of IL-21.

**Table 2:** Common adverse events* and laboratory abnormalities in phase 2 patients treated with IL-21 (30 mcg/kg) plus sorafenib (starting at 400 mg twice daily)

Adverse event preferred term* [N = 33 unless noted]	**Any grade n (%)**	**Grade 3 n (%)**	**Grade 4 n (%)**
Rash^†^	31 (94)	9 (29)	—
Fatigue	23 (70)	2 (6)	1 (3)
Diarrhea	20 (61)	2 (6)	—
Pyrexia	20 (61)	—	—
Chills	18 (55)	—	—
Palmar-plantar erythrodysaesthesia (hand-foot syndrome)	18 (55)	8 (24)	—
Alopecia	15 (45)	—	—
Vomiting	14 (42)	2 (6)	
Influenza-like illness	13 (39)	—	—
Headache	12 (36)	—	—
Nausea	12 (36)	2 (6)	
Pruritus	12 (36)	—	—
Arthralgia	10 (30)	—	—
Pain in extremity	10 (30)	1 (3)	—
**Laboratory abnormality**^§^	**Any grade**	**Grade 3 n (%)**	**Grade 4 n (%)**
Lymphopenia	32 (97)	16 (48)	7 (21)
Hypophosphatemia	27 (82)	17 (52)	2 (6)
Lipase high (n = 31)	16 (52)	7 (23)	1 (3)
Hyponatremia (n = 22)	22 (100)	5 (23)	—
Platelets low	27 (82)	6 (18)	1 (3)
Hyperuricemia (n = 32)	16 (50)	—	7 (22)
Platelets low	27 (82)	6 (18)	1 (3)
AST high	22 (67)	6 (18)	—
ALT high	22 (67)	5 (15)	—
Leukocytes low	20 (61)	4 (12)	—
Neutrophils low	18 (55)	1 (3)	3 (9)
Hypokalemia (n = 9)	9 (100)	1 (11)	—

*Adverse events occurring in at least 30% of patients treated are included here.

^†^Includes any rash preferred term.

^§^Laboratory abnormalities occurring in at least 50% of patients treated are included. Those occurring in <50% of patients included predominantly grade 1 or 2 electrolyte changes of no significant clinical consequence.

**Figure 1:**
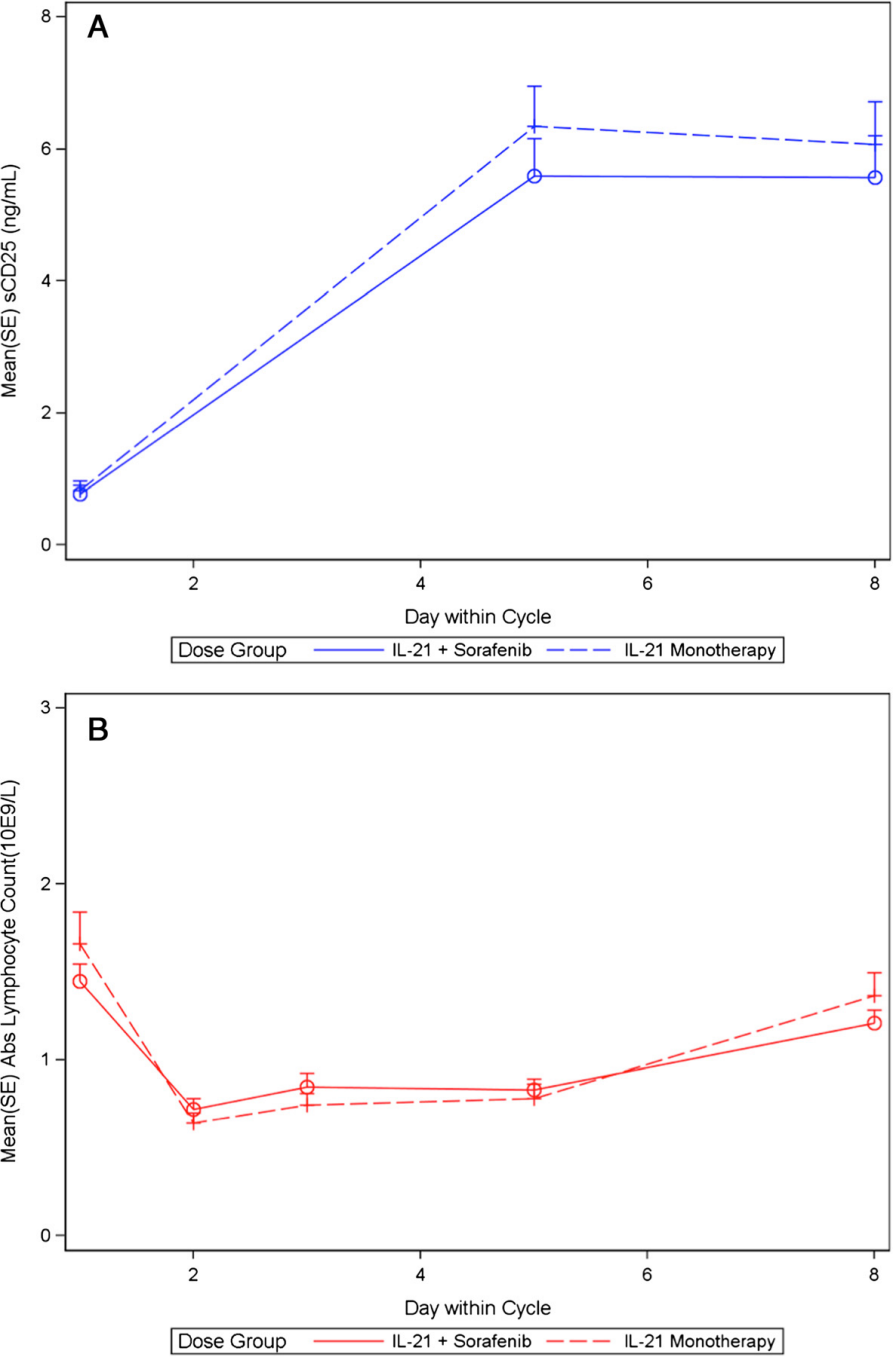
**Pharmacodynamic effects of IL-21 at the 30 mcg/kg dose, as monotherapy (previous study [**[22]**]) *versus *in combination with sorafenib (current study). (A)** Induction of serum sCD25, **(B)** Changes in peripheral blood absolute lymphocyte count (ALC).

The most common laboratory abnormalities in phase 2 patients included cytopenias, electrolyte abnormalities, and elevated hepatic transaminases. These were mostly grade 1 or 2 and were transiently observed during IL-21 treatment weeks. Transient lymphopenia was observed during the IL-21 administration weeks with rapid recovery afterwards, a pattern similar to the observations from IL-21 monotherapy study (Figure 1) [22]. Grade 3 hypophosphatemia, although common, was typically asymptomatic and responded well to oral supplementation. Adverse effects on renal and hepatic function were mostly mild and transient, although reversible grade 3 elevations in creatinine and hepatic transaminases occurred sporadically.

The majority of patients (70%) required a reduction in the sorafenib dose mostly due to skin rash and HFS. After reduction in sorafenib dose, most patients tolerated the combination treatment well without a recurrence of these toxicities. The IL-21 dose was reduced in 3 patients due to myalgias, pancreatitis, and rash, respectively. No treatment-related deaths were observed in this study.

### Pharmacokinetics, pharmacodynamics and immunogenicity

Exposure parameters for IL-21 increased with dose and did not appear to change significantly with repeat dosing. The mean overall exposure based on AUC_0-t_ after a single and repeated doses of 30 mcg/kg IL-21 in combination with sorafenib was 188 (34% CV) and 226 (37% CV) h*ng/mL respectively. The corresponding mean half-life estimates were 1.82 (23% CV) and 1.95 (13% CV) hours. These PK parameter estimates are similar to those observed with IL-21 monotherapy [22]. As IL-21 PK did not change with time, the addition of oral doses of sorafenib does not appear to affect the PK of IL-21. Single dose sorafenib exposure parameters (C_max_, AUC) in the presence of IL-21 appear comparable to reported values for single-agent sorafenib (data not shown) [32]. The effect of IL-21 on sorafenib repeat-dose PK could not be determined due to the frequency of sorafenib dose reductions.

Soluble CD25 (sCD25; α-subunit of the IL-2 receptor) is cleaved from T and NK cells on activation [24]. While this study did not specifically assess cytotoxic function of CD8 T- or NK- cells (previously evaluated in other IL-21 trials [21,33]), the serum levels of sCD25 were measured at multiple time points to broadly assess T- and NK- cells immune activation from IL-21, as described previously [21,22]. The serum concentration of sCD25 increased in all dose cohorts following IL-21 dosing. In addition, sCD25 induction following dosing with 30 mcg/kg IL-21 in combination with sorafenib was consistent with previous observations with IL-21 monotherapy (Figure 1), suggesting that sorafenib does not interfere with the pharmacological effects of IL-21 [21,22].

Neutralizing anti–IL-21 antibodies were detected in 3 patients. Two of these 3 patients developed infusion reactions characterized as transient flushing, chills, and mild hypotension; both patients continued to receive IL-21 after pre-medication with antihistaminics and acetaminophen. While the effect of these antibodies on IL-21 PK was not analyzed, the development of these antibodies did not appear to affect clinical responses; one patient developed a PR after seroconversion, another patient continued with SD after seroconversion, and the third patient had PD during the same cycle as seroconversion. The clinical significance of the anti–IL-21 antibodies, which were noted in the phase 1 monotherapy trial as well, remains unclear [22].

### Antitumor effect

**Figure 2:**
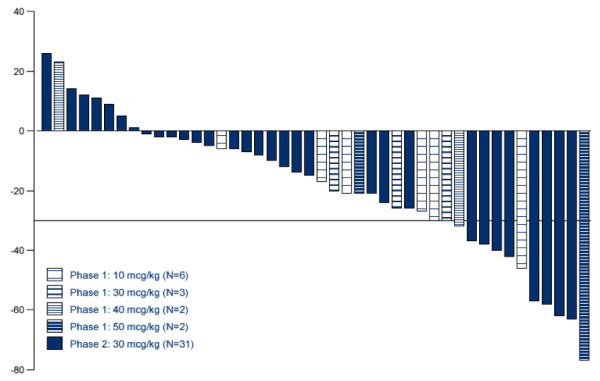
**Maximum tumor reduction (per Independent radiologic review) in study patients receiving IL-21 (10-50 mcg/kg) plus sorafenib (starting at 400 mg twice daily).** The maximum percent change from baseline in the sum of the longest diameters of target lesions per RECIST v1.0 is depicted for the study patients. (NOTE: Two patients from phase 2 are not included due to clinical progressive disease in one patient and withdrawal from the study due to an AE prior to the restaging evaluation in another patient).

Antitumor activity was observed at all dose levels of IL-21 in combination with sorafenib, with the majority of patients experiencing shrinkage of the target tumor lesions per RECIST (Figure 2). Thirteen phase 1 patients completed at least 1 full treatment course and were evaluable for response assessment; 3 of these 13 patients (23%) had a PR and 9 of 13 patients (69%) had SD by independent radiologic review (Figure 2). In the phase 2 portion of the study, 7 of the 33 patients (21%) [95% CI: 9, 38.9] had a confirmed PR and 20 of 33 patients (61%) [95% CI: 42.1, 77.1] had SD by independent review; DCR was 82% [95% CI: 64.5, 93] (Table 3, Figure 2). The characteristics of responding patients are shown in Table 4; responses were seen regardless of the site of disease or the type of prior therapy. The majority of responders had received prior targeted therapies including VEGFR-TKIs and/or mTOR inhibitors. Median PFS was 5.6 months. Two patients had durable partial responses (near CRs with persistent small residual masses) that were ongoing at 41+ months and 30+ months after treatment initiation; there had been no growth in the small residual masses several months after cessation of both IL-21 and sorafenib.

**Table 3:** Best overall response (per RECIST) for patients treated in Phase 2 with IL-21 (30 mcg/kg) plus sorafenib (starting at 400 mg twice daily)

**RECIST response**	**Investigator assessment (N = 33)**	**Independent review (N = 33)**
Complete response	0	0
Partial response	6 (18%)	7 (21%)
Stable disease	22 (67%)	20 (61%)
Progressive disease	4 (12%)	4 (12%)
Unavailable*	1 (3%)	2 (6%)

*Includes 1 subject who discontinued for toxicity prior to disease reassessment by both investigator and independent review and 1 subject with progressive disease on imaging not sent for independent review. All objective responses were confirmed on a subsequent imaging study.

**Table 4:** Characteristics of Phase 2 patients who had objective responses after treatment with IL-21 plus sorafenib

**Subject**	**Best response (Independent assessment)**	**Site(s) of disease**	**Prior therapy(ies)**
1021	PR	Liver, LNs	Sunitinib, Temsirolimus
1027	PR	Lung, LNs, pancreas, bone	Pazopanib
2036	PR	Kidney, adrenal, lungs	Temsirolimus
2041	PR	Liver, tongue	Cediranib
2042	PR	Liver, lung	Sunitinib
2045	PR	LNs, liver	High-dose IL-2
2046	PR	Pancreas, lung, LNs, bone, peritoneum	Temsirolimus

*Abbreviations*: *IL-2*, interleukin-2; *PR*, partial response; *LNs*, lymph nodes.

Baseline characteristics were evaluated to identify factors predictive of positive IL-21 response. Baseline VEGF levels have been suggested to predict anti-tumor response to high-dose IL-2 and to VEGFR-targeted therapies [34,35]. In this study, no significant association between baseline VEGF levels and clinical efficacy endpoints (ORR or PFS) was observed (Additional file 1: Table S1). In addition, neither baseline sCD25 nor IL-21-mediated sCD25 induction were found to correlate significantly with clinical efficacy (Additional file 1: Table S2A and S2B, respectively).

## Discussion

This phase 1/2 trial defines the MTD, safety and activity of an outpatient treatment regimen that includes IL-21, a cytokine with unique immunostimulatory properties, in combination with sorafenib, a VEGFR-TKI, in patients with mRCC. The combination of IL-21 at 30 mcg/kg and sorafenib appears to be safe with appropriate dose reductions in sorafenib and to have antitumor activity in mRCC patients who have failed prior targeted and/or cytokine therapies.

The optimal dose of IL-21 in combination with the standard dose of sorafenib (starting at 400 mg twice daily) was identified as 30 mcg/kg/day. In general, AEs observed in this study were consistent with toxicities associated with either agent alone. The most common toxicities included constitutional, dermatologic and gastrointestinal symptoms. Dermatologic toxicity was the predominant reason for sorafenib dose modifications. Grade 3 skin rash was the DLT in phase 1 patients. Rash was also observed in 94% (grade 3 in 29%) of phase 2 patients, a higher proportion than expected with either drug administered alone [2,22,36]. Similarly, HFS was observed in 55% of phase 2 patients; the proportion of grade 3 HFS (24%) was higher than that observed with sorafenib monotherapy [2,36]. The rate of sorafenib dose-reductions in this study (70%) is somewhat higher as compared to sorafenib monotherapy trials (13–52%) [2,36,37]. Reassuringly, most patients who required dose-reductions in sorafenib in our trial tolerated the combination treatment well at reduced doses of sorafenib (usually 400 mg/day) without recurrence of severe toxicity. Also, there were no unexpected cumulative toxicities with administration of repeat courses of IL-21 plus sorafenib. Our study supports the feasibility of cytokine therapy using IL-21 in patients previously treated with VEGFR-TKIs, while there have been safety concerns about using other cytokines such as HD IL-2 in such patients [38].

While interpreting the efficacy results from this non-randomized phase 1/2 study, it is important to keep in mind the limitations of small sample size and selection bias in phase 2 trials. Similarly, caution should be exercised in any comparison across trials due to differences in sample size, patient population and study methods. The clinical activity of targeted agents in mRCC is consistently lower in second, or subsequent lines of therapy compared to first-line therapy suggesting an unmet need for this population. Everolimus, the FDA-approved agent for patients who have failed VEGFR-TKIs, was associated with an ORR of 1% and a median PFS of 4 months [5]. The current study’s ORR of 21% (including two patients with durable responses) and the median PFS of 5.6 months are encouraging in this pretreated (mostly with agents targeting the VEGF pathway) patient population. The antitumor activity of this combination compares favorably to the historical activity of sorafenib monotherapy. In a phase 3 study of sorafenib in pretreated (mostly with cytokines) mRCC patients, the ORR was low (2% by independent radiologic review) with no CRs [2]. Similarly, in various studies of sorafenib in patients who had previously received VEGF-targeted therapies, response rates have been low (0%–9.6%) with a modest median PFS or time-to-progression (around 4 months) [37,39-43]. Although it is not possible to discern the relative contributions of IL-21 and sorafenib to the overall antitumor activity in this single-arm study, it is plausible that IL-21 contributed to the activity of the combination, given the modest ORR and PFS generally seen with sorafenib monotherapy in mRCC patients who have previously been treated (especially with VEGF-targeted therapies). Also, while the ORR in this trial appears similar to that seen with the IL-21 monotherapy trial, the small sample sizes and the differences in patient population in the two studies preclude a direct comparison. The majority of the patients with an objective response in our trial had previously received targeted therapies, while most patients in the phase 1 IL-21 monotherapy study were either treatment-naïve or previously treated with cytokines. The durability of (near-complete) responses in two patients that persisted despite cessation of therapy highlight the potential of cytokine immunotherapy to significantly advance outcomes in a subset of mRCC patients. However, the infrequent occurrence of durable responses, the desired outcome, also underscores the importance of identifying predictive biomarkers in future trials.

Previous efforts to combine immunotherapy with VEGFR-TKI in patients with RCC have yielded conflicting results. The results of our trial are in contrast to another trial that tested the combination of IL-21 with sunitinib, also a VEGFR-TKI [44]. That trial was discontinued after the observation of severe hematologic DLTs at the IL-21 dose of 10 mcg/kg in combination with standard dose of sunitinib. However, sunitinib has proven to be a challenging drug to combine with cytokines or other therapies due to its toxicity profile [45,46]. Other VEGFR-TKIs may be better suited for combination with cytokines. Two studies investigated the combination of sorafenib with standard-dose IFN in previously untreated patients with good performance status; although efficacy results were encouraging, the majority (65–79%) of patients required IFN dose-reductions with a high treatment-discontinuation rate (25%) due to toxicities [47,48]. Another study compared sorafenib plus low-dose IFN combination with sorafenib monotherapy and found no difference in efficacy between the two arms, although there was less toxicity in the combination arm than that observed in the above-mentioned trials using standard-dose IFN [49]. In our study, the MTD of IL-21 in combination with sorafenib is the same as the monotherapy dose of IL-21; further, IL-21 dose-reductions were uncommon, allowing for full immunotherapeutic effects of the agent. Lymphocyte activation by IL-21, as determined by sCD25 levels, appears to be retained in the presence of sorafenib. Hence, IL-21 may represent a suitable immunotherapy for further exploration of combination strategies in mRCC, especially with the emerging more selective VEGFR-TKIs (such as axitinib) and with other approaches designed to stimulate the immune system. Trials investigating the combination of IL-21 with other immunotherapy agents, such as ipilimumab and anti-PD-1 antibody, in patients with solid tumors including mRCC are also ongoing (http://www.clinicaltrials.gov; NCT01489059 and NCT01629758 respectively).

Some preclinical studies have associated sorafenib, but not sunitinib, with relative impairment of the NK-cell effector function [50] and of the dendritic cells and adaptive immune responses [51]. However, the clinical significance of these preclinical findings has been unclear. Sorafenib therapy has not been associated with increased risk of infections, which would have supported a drug’s immunosuppressive potential, in the major clinical trials [2]. In the preclinical study of IL-21 plus sorafenib in the murine RenCa model, sorafenib did not inhibit the effects of IL-21 on CD4+ or CD8+ T cell proliferation, NK cell activation, or antibody-dependent cellular cytotoxicity, and led to improved tumor shrinkage and survival time as compared to either therapy alone [29]. Similarly, the combination of sorafenib with Interleukin-2 in murine studies did not show any significant inhibitory effects of sorafenib on IL-2 induced NK-cell expansion [25]. While the paucity of well-defined RCC antigens/biomarkers limits our ability to rigorously assess the effects of sorafenib on IL-21 induced tumor specific immune responses in this study, the data on sCD25 levels and the lymphocyte counts suggest that sorafenib did not interfere with the pharmacological effects of IL-21.

## Conclusions

Combination therapy with IL-21 and sorafenib has antitumor activity with acceptable safety in previously treated mRCC patients. Given its unique immunostimulatory properties, antitumor activity, and tolerability in an outpatient regimen, IL-21 may also be suitable for combination with other antiangiogenic and immunomodulatory therapies. Such combinations may increase the efficacy of existing therapies and lead to improved patient outcomes.

## Methods

### Study treatment and design

This was a phase 1/2, open-label, multicenter study of IL-21 given in combination with sorafenib to patients with mRCC. Sorafenib was administered at the US FDA-approved dosing schedule of 400 mg orally twice daily starting on day 1 with dose modifications allowed per the package insert. Recombinant IL-21 [ZymoGenetics (now Bristol-Myers Squibb), Seattle, WA] was administered by rapid intravenous (IV) injection daily on days 1–5 (week 1) and 15–19 (week 3) of a 7-week treatment course, in an outpatient treatment setting. Restaging radiologic evaluations were performed during the seventh week of each treatment course. Patients with stable disease (SD) or better were eligible for retreatment with additional courses of IL-21 plus sorafenib.

In the phase 1 portion, a 3 + 3 dose-escalation design was used to estimate the maximum tolerated dose (MTD) of IL-21 in combination with the standard dose of sorafenib. Four dose levels of IL-21 (10, 30, 40 and 50 mcg/kg) were tested in cohorts of up to 6 evaluable patients per dose, starting at the 10 mcg/kg dose level. Even though the MTD of IL-21 monotherapy was 30 mcg/kg in the phase 1 monotherapy trial, the only patient treated with 50 mcg/day dose in that trial had transient grade 3 neutropenia that did not recur with re-treatment [22]. Hence, two dose levels of IL-21 above 30 mcg/kg were included in the current study.

The phase 2 portion of the study further evaluated the safety and antitumor activity of IL-21 administered at the MTD in combination with sorafenib in mRCC patients receiving second- or third-line treatment.

### Patients

Eligibility requirements included mRCC of predominantly clear-cell histology; age ≥ 18 years; measurable disease per Response Evaluation Criteria in Solid Tumors (RECIST) version 1.0 [52]; life expectancy > 6 months; Eastern Cooperative Oncology Group (ECOG) performance status 0 or 1; prior nephrectomy; no brain metastases; no uncontrolled hypertension; and adequate renal, hepatic and hematologic function. Prior systemic therapy for mRCC was required for phase 2 patients, but no more than 2 prior systemic therapeutic regimens were allowed; prior IL-21 or sorafenib administration was not allowed. Institutional review boards of participating centers approved the protocol, and patients gave written informed consent before study-specific procedures began.

### Safety and efficacy assessments

Toxicities were evaluated using National Cancer Institute’s Common Terminology Criteria for Adverse Events (CTCAE) version 3.0. Dose-limiting toxicity (DLT) was designated during treatment course 1 and was defined as any treatment-related clinical adverse event (AE) ≥ grade 3 (except fatigue, fever or transient rigors, nausea or vomiting without antiemetic therapy) or any treatment-related grade 4 (or grade 3 lasting >3 days) laboratory abnormalities (except lymphopenia, leukopenia, transient neutropenia not associated with infection, asymptomatic electrolyte abnormalities and asymptomatic elevations in amylase/lipase). Safety endpoints included incidence and severity of adverse events and clinical laboratory abnormalities.

For antitumor activity assessment, results from restaging radiologic evaluations were categorized per RECIST version 1.0 as CR, partial response (PR), SD or progressive disease (PD) [52]. Both investigator and independent radiology review were conducted for all patients. Efficacy endpoints included ORR (defined as the rate of PR + CR at the time of best response), disease control rate (defined as the rate of SD + PR + CR at the time of best response) and progression free survival (PFS).

### Pharmacokinetics, pharmacodynamics and immunogenicity

Serum and plasma samples were collected at select time points for evaluation of IL-21 and sorafenib pharmacokinetics (PK), respectively. IL-21 concentration was determined using a validated ELISA (ZymoGenetics, Seattle, WA); the lower limit of quantification of this assay was 0.112 ng/mL. Sorafenib concentration was determined utilizing liquid chromatography with the tandem mass spectrometric detection method (Covance Bioanalytical Services, IN). C_max_ (maximum observed concentration), AUC_0-t_ (area under the concentration versus time curve from time zero to the last measurable timepoint), and t_1/2,λz_ (terminal half-life) were estimated using WinNonlin Professional v5.2.1 software. Due to the sparse sampling scheme, reported t_1/2,λz_ values should be interpreted with caution.

Serum samples to determine soluble CD25 (sCD25) concentration using a validated enzyme-linked immunosorbent assay (ELISA) (ZymoGenetics, Seattle, WA) were drawn at select time points during course 1. Baseline serum and plasma VEGF levels were determined using a validated immunoassay (Quest Inc., Valencia, CA). The relationships of baseline VEGF and sCD25 levels and that of change in sCD25 levels to clinical efficacy endpoints (ORR and PFS) were explored.

Serum specimens were collected at select time points to screen for IL-21 binding antibodies using ELISA (ZymoGenetics, Seattle, WA); samples containing IL-21 specific antibodies were evaluated for neutralizing activity by a cell-based bioassay.

### Statistical analysis

Based on the binomial distribution, it was determined that a sample size of 30 subjects in the phase 2 portion of the study would provide approximately 95% probability of observing a relevant safety event in one or more subjects if the true population incidence rate was 10% or greater. Given the early phase of this study, formal assessments of power for efficacy endpoints were not conducted.

Kaplan-Meier estimates for median PFS were computed with the earliest assessment of progression (by investigator or independent review) treated as time of progression. SAS version 9.1 was used to perform all analyses.

The association between baseline levels of VEGF and sCD25, as well as sCD25 induction, with outcomes of clinical efficacy was explored using a series of Cox regression (PFS) and logistic regression (ORR) models (both raw and log-transformed values of each biomarker were analyzed).

### Results from this study have been presented in part at the following conferences

AACR 2007 – poster (Bhatia)

EORTC 2008 – poster (Flaig)

iSBTc 2008 – presentation (Bhatia)

ASCO 2009 – poster (Bhatia)

## Competing interest

This study was supported by ZymoGenetics (Bristol-Myers Squibb), Seattle, WA. The authors have reported the following:

SB: Research funding (Zymogenetics, BMS)

BC: Research funding (Zymogenetics); Uncompensated Advisory Role (Zymogenetics)

MSE: Research funding (Zymogenetics)

MG: Research funding (Zymogenetics)

EIH: Research funding (Zymogenetics)

WHM: None

IP: None

DIQ: Compensated Advisory Role (Bayer, Aveo, Onyx, Genentech, Pfizer, Novartis); Honoraria (Bayer, Onyx)

TWG: Honoraria (Amgen); Research funding (Genentech, Bayer/Onyx, Pfizer, GSK, Novartis)

PVV: None

KBB: Previously Employed at Zymogenetics

JAF: Previously Employed at Zymogenetics

RB: Currently employed at Zymogenetics

NH: Previously Employed at Zymogenetics

SS: Previously Employed at Zymogenetics

JAT: Research funding (Zymogenetics, BMS); Compensated Advisory Role (Zymogenetics)

## Authors’ contributions

Conception and Design: BC, DIQ, KBB, JAF, NH, SS, JT. Collection and Assembly of data: SB, BC, MSE, MG, EIH, WHM, IP, DIQ, TWF, PVV, NH, JT. Data Analysis and interpretation: SB, BC, MSE, MG, EIH, WHM, IP, DIQ, TWG, PVV, KBB, JAF, RB, NH, SS, JT. Manuscript writing and final approval of manuscript: SB, BC, MSE, MG, EIH, WHM, IP, DIQ, TWG, PVV, KBB, JAF, RB, NH, SS, JT. All authors read and approved the final manuscript.

## Acknowledgements

The authors would like to thank Audrey Mollerup (University of Washington) and Janet Kramer (Zymogenetics) for their contributions to the conduct of this study and to the development of IL-21 in general. Most importantly, the patients and their families who participated in this study deserve a special mention for entrusting us with their care.

## Supplementary Material

Additional file 1: Table S1 Baseline serum VEGF levels, overall and by ORR categories. There does not appear to be any trend in mean or median VEGF by RECIST responses observed in the non-transformed serum VEGF values. Similar results were seen for the log-transformed serum VEGF values (data not shown). **Table S2A** Baseline serum sCD25 levels, overall and by ORR categories. There does not appear to be any trend in mean or median sCD25 by RECIST responses observed in the non-transformed serum sCD25 values. Similar results were seen for the log-transformed serum sCD25 values (data not shown). **Table S2B** Change from Baseline serum sCD25 levels, overall and by ORR categories. There does not appear to be any trend in mean or median Change from Baseline serum sCD25 levels by RECIST responses observed in the non-transformed serum sCD25 values. Similar results were seen for the log-transformed serum sCD25 values (data not shown).  Click here for file
