# Supplementary material for: Recombinant interleukin-21 plus sorafenib for metastatic renal cell carcinoma: a phase 1/2 study
Source: J Immunother Cancer. 2014 Jan 27;2:2. doi: 10.1186/2051-1426-2-2 (PMC4019894; doi:10.1186/2051-1426-2-2)
Supplement: Additional file 1: Table S1 — Baseline serum VEGF levels, overall and by ORR categories. There does not appear to be any trend in mean or median VEGF by RECIST responses observed in the non-transformed serum VEGF values. Similar results were seen for the log-transformed serum VEGF values (data not shown). Table S2A Baseline serum sCD25 levels, overall and by ORR categories. There does not appear to be any trend in mean or median sCD25 by RECIST responses observed in the non-transformed serum sCD25 values. Similar results were seen for the log-transformed serum sCD25 values (data not shown). Table S2B Change from Baseline serum sCD25 levels, overall and by ORR categories. There does not appear to be any trend in mean or median Change from Baseline serum sCD25 levels by RECIST responses observed in the non-transformed serum sCD25 values. Similar results were seen for the log-transformed serum sCD25 values (data not shown). [file 2051-1426-2-2-S1.docx]

**Additional file**

Table S1. Baseline serum VEGF levels, overall and by ORR categories. There does not appear to be any trend in mean or median VEGF by RECIST responses observed in the non-transformed serum VEGF values. Similar results were seen for the log-transformed serum VEGF values (data not shown).

| Baseline Serum VEGF (pg/ml) | | | | | |
| --- | --- | --- | --- | --- | --- |
|  | RECIST Criteria | | | | Overall |
|  | PR | PRu | SD | PD |  |
| n | 6 | 3 | 23 | 5 | 37 |
| Mean | 382.6 | 269.9 | 566.5 | 435.6 | 494.9 |
| std. deviation | 233.675 | 191.8 | 431.8 | 258.2 | 375.6 |
| Median | 300.3 | 238.7 | 472.8 | 517.8 | 397.1 |
| Min, Max | 189.1, 838.6 | 95.6, 475.4 | 80.6, 1609.5 | 80.2, 699.7 | 80.2, 1609.5 |

PRu = Partial Response, Unconfirmed

Table S2A. Baseline serum sCD25 levels, overall and by ORR categories. There does not appear to be any trend in mean or median sCD25 by RECIST responses observed in the non-transformed serum sCD25 values. Similar results were seen for the log-transformed serum sCD25 values (data not shown).

| Baseline Serum sCD25 ng/ml | | | | | |
| --- | --- | --- | --- | --- | --- |
|  | RECIST Criteria | | | | Overall |
|  | PR | PRu | SD | PD |  |
| n | 9 | 3 | 26 | 5 | 43 |
| Mean | 0.853 | 0.616 | 0.872 | 0.780 | 0.839 |
| std. deviation | 0.456 | 0.313 | 0.648 | 0.537 | 0.572 |
| Median | 0.740 | 0.648 | 0.772 | 0.552 | 0.712 |
| Min, Max | 0.320, 1.510 | 0.288, 0.912 | 0.252, 3.120 | 0.472, 1.730 | 0.252, 3.120 |

PRu = Partial Response, Unconfirmed

Table S2B. Change from Baseline serum sCD25 levels, overall and by ORR categories. There does not appear to be any trend in mean or median Change from Baseline serum sCD25 levels by RECIST responses observed in the non-transformed serum sCD25 values. Similar results were seen for the log-transformed serum sCD25 values (data not shown).

| Change From Baseline Serum sCD25 ng/ml  (Course 1 Cycle 1 Day 5 – Baseline) | | | | | |
| --- | --- | --- | --- | --- | --- |
|  | RECIST Criteria | | | | Overall |
|  | PR | PRu | SD | PD |  |
| n | 9 | 3 | 25 | 4 | 41 |
| Mean | 6.256 | 8.581 | 5.924 | 6.040 | 6.203 |
| std. deviation | 2.048 | 2.519 | 3.545 | 2.650 | 3.114 |
| Median | 7.130 | 7.202 | 5.254 | 5.426 | 5.932 |
| Min, Max | 2.714, 8.360 | 7.052, 11.488 | 1.134, 16.388 | 3.538, 9.770 | 1.134, 16.388 |

PRu = Partial Response, Unconfirmed
